# Displacement of submacular hemorrhage secondary to age-related macular degeneration with subretinal injection of air and tissue plasminogen activator

**DOI:** 10.1038/s41598-022-26289-6

**Published:** 2022-12-22

**Authors:** Maasa Ogata, Hideyasu Oh, Ai Nakata, Ayaka Doi, Hiroki Nakayama, Mariko Hasegawa, Miou Hirose

**Affiliations:** 1 Department of Ophthalmology, Hyogo Prefectural Amagasaki General Medical Center, Higashinaniwa-Cho 2-17-77, Amagasaki, Hyogo 660-8550 Japan; 2 Department of Ophthalmology, Mitsubishi Kyoto Hospital, Kyoto, Japan; 3 Department of Ophthalmology, Kansai Medical Universal Hospital, Osaka, Japan

**Keywords:** Macular degeneration, Retinal diseases

## Abstract

Submacular hemorrhage (SMH) can lead to devastating visual loss in patients with age-related macular degeneration. We retrospectively evaluated the surgical outcomes of vitrectomy with subretinal injection of tissue plasminogen activator, bevacizumab, and air in 13 cases. Visual prognosis, anatomical results obtained with optical coherence tomography (OCT), and their correlations were investigated. We analyzed OCT parameters including SMH height, pigment epithelial detachment (PED) height and width, and status of ellipsoid zone (EZ) line. Complete displacement of SMH was achieved in 12 eyes. At 3 months post-surgery, best-corrected visual acuity (BCVA) and SMH height exhibited significant improvements (P < 0.01). In eyes with preoperative SMH height < 300 µm and a detectable EZ line, BCVA was significantly improved at as early as 1 month, whereas the remaining eyes exhibited visual improvements only at 3 months. Postoperative BCVA positively correlated with preoperative BCVA (r = 0.86, P < 0.005), and negatively correlated with SMH size (r = 0.69, P < 0.01) and PED height (r = 0.58, P < 0.05) and width (r = 0.67, P < 0.05). Multivariate analyses confirmed preoperative BCVA as the predominant factor associated with postoperative BCVA (β = 1.093, P < 0.05). In conclusion, significant improvements in BCVA and anatomical findings can be achieved with our reported surgical technique. Preoperative OCT findings may influence the duration required for visual improvements.

## Introduction

Submacular hemorrhage (SMH) is a pathological condition that can lead to devastating visual loss. SMH occurs in various retinal diseases, most commonly age-related macular degeneration (AMD), presumed retinal arterial microaneurysm, ocular histoplasmosis, trauma, and high myopia^1^. In untreated SMH due to AMD, visual outcomes are often poor^2,3^, and initial thickness and size of the hemorrhage are reported to negatively influence visual outcomes^4,5^. Blood clots in the subretinal area induce damage to photoreceptor segments via various mechanisms, including mechanical damage to the outer segments by clot contraction, tearing of photoreceptors by fibrin, and iron toxicity^6,7^.

Various approaches for surgical management of massive SMH have been reported, including intravitreal injection of gas with or without tissue plasminogen activator (t-PA), pars plana vitrectomy (PPV) with or without t-PA, and intravitreal injection of anti-vascular endothelial growth factor (VEGF) antibodies. PPV and subretinal injection of t-PA were first reported by Haupert et al.^8^, followed by a larger study conducted by Olivier et al.^9^ The results of these studies revealed that this method was more effective compared to vitrectomy alone. Moreover, Hillenkamp et al. reported that vitrectomy with subretinal injection of t-PA was more effective in terms of complete displacement of SMH compared to vitrectomy with intravitreal injection of t-PA^10^.

We previously reported the surgical results of PPV with subretinal injection of t-PA^11^ alone. In that study, liquefied SMH was evacuated as much as possible during surgery. The mean improvement in best-corrected visual acuity (BCVA) at 3 months postoperatively was 0.54 in logarithm of the minimum angle of resolution (log MAR) and the rate of SMH displacement was 100%. In this study, subretinally injected t-PA was also removed during surgery, which might explain the relatively good outcomes compared to other previous studies that did not employ this step^8–10^. However, the prognosis can be improved further by other surgical techniques still remains to be explored.

Martel et al. recently reported a surgical technique of delivering subretinal t-PA with or without bevacizumab and air to facilitate displacement of SMH^12^. With this technique, subretinal air was expected to localize at the submacular space due to its buoyancy, thus protecting the macula from being re-occupied by the original hemorrhage. However, the specific effects of this technique on anatomical results analyzed using optical coherence tomography (OCT) findings have yet to be investigated. In this study, we present the surgical outcomes of patients treated with this technique and their relationship to anatomical findings documented on OCT.

## Results

### Patient characteristics

This study included 13 eyes of 13 patients. All patients had SMH secondary to exudative AMD. The diagnosis was typical AMD in 6 eyes, polypoidal choroidal vasculopathy (PCV) in 6 eyes, and retinal angiomatous proliferation (RAP) in 1 eye. During the studied period, one patient with less than 1 disc diameter of SMH were excluded from this study and treated with intravitreal injection of aflibercept (IVA). The baseline characteristics of all cases are summarized in Table 1. The mean age of patients was 77.2 years old (range, 66–88 years). The average period between symptom onset and surgery was 10 days (range, 2–30 days). Three patients (23.0%) received anticoagulant medications. Of the 13 patients, 10 (76.9%) received postoperative anti-VEGF injections to further reduce residual fluid and/or hemorrhage located in the intraretinal or subretinal space. Patients 1 and 2 received consecutive intravitreal IVA monotherapy before surgery. Patient 3 received consecutive intravitreal ranibizumab (IVR) followed by consecutive IVA before surgery. Patient 4 was treated with photodynamic therapy (PDT) twice and IVA 8 times. Patient 8 received PDT thrice, IVA twice, and IVR 8 times before surgery. Patient 10 received consecutive IVR monotherapy. Patient 12 received PDT twice and focal laser photocoagulation 10 years prior to surgery. The 6 remaining patients were treatment-naïve before surgery.

**Table 1:** Demographic data.

Patient No	Age (years)	Diagnosis	Submacular hemorrhage duration (days)	Size of SMH (disc size)	Treatments before surgery	Anticoagulant medication
1	77	PCV	9	5.2	+	−
2	76	AMD	Unknown	5.8	+	+
3	80	PCV	3	3	+	+
4	66	AMD	2	5	+	−
5	90	PCV	8	5.2	−	−
6	71	PCV	30	3.5	−	−
7	76	AMD	2	3.5	−	−
8	69	AMD	30	5.5	+	+
9	71	AMD	13	8	−	−
10	88	RAP	3	4.7	+	−
11	83	PCV	7	3.5	−	−
12	84	PCV	2	3	+	−
13	72	AMD	14	2.3	−	−

## Preoperative OCT findings

Preoperative mean SMH height and mean height and width of pigment epithelial detachment (PED) were 351.3 ± 69.7 µm, 390.2 ± 92.5 µm, and 3094.5 ± 561.6 µm, respectively (Table 2). Preoperative ellipsoid zone (EZ) of the fovea was complete in 4 eyes, incomplete in 2 eyes, and absent in 7 eyes. Mean PED number was 1.23 ± 0.17 within the 8.9 mm × 6.0 mm area of the retina map.

**Table 2:** Preoperative OCT findings.

Patient no	Height of SMH (µm)	Height of PED (µm)	Width of PED (µm)	Number of PED	Detection of EZ
1	179	279	5025	2	Absent
2	495	725	4715	2	Absent
3	258	520	2923	1	Complete
4	978	0	0	0	Incomplete
5	199	849	4686	1	Incomplete
6	88	421	3848	2	Absent
7	172	65	442	1	Complete
8	324	138	4869	1	Absent
9	174	1986	3524	2	Complete
10	180	253	5291	1	Absent
11	604	0	0	1	Absent
12	588	419	3962	1	Absent
13	328	318	943	1	Complete

### Visual outcomes

**Figure 1:**
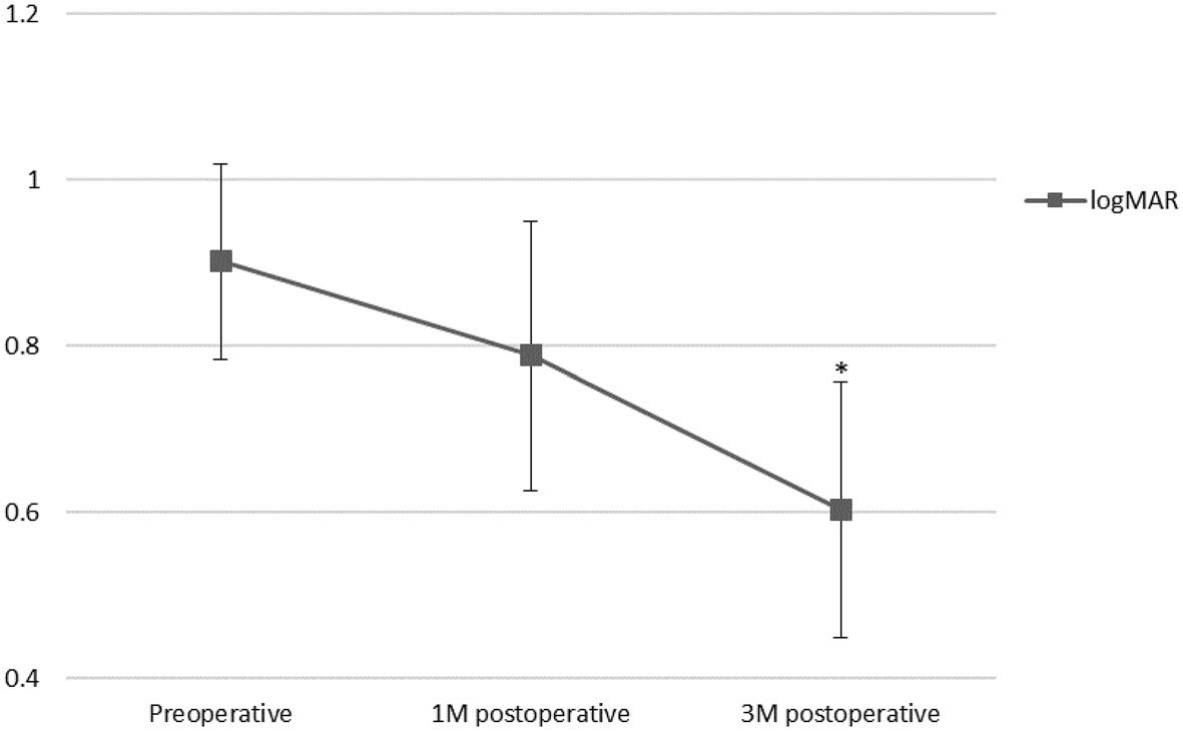
Mean BCVA of all patients. *P < 0.01.

Mean preoperative visual acuity was 20/160 (0.90 ± 0.12 in log MAR, which improved slightly to 20/125 (0.79 ± 0.16 in log MAR) at 1 month (P > 0.05) and improved significantly to 20/80 (0.60 ± 0.15 in log MAR) at 3 months (P < 0.05) (Fig. 1). Compared with preoperative visual acuity, postoperative log MAR at 1 month improved or worsened by at least 0.1 log MAR in 7 eyes and 2 eyes, respectively. The remaining 4 eyes exhibited a change in log MAR of less than 0.1. At 1 month after surgery, two cases presented with worsening of BCVA due to recurrent SMH (patient 2) and postoperative retinal detachment (patient 9). Similarly, postoperative BCVA at 3 months improved in 9 eyes, worsened in 1 eye, and remained unchanged in 3 eyes (Table 3).

**Table 3:** Changes in BCVA and postoperative EZ status.

Patient no	Preoperative BCVA (Snellen)	1 M postoperative BCVA (Snellen)	3 M postoperative BCVA (Snellen)	Detection of 3 M postoperative EZ	Postoperative treatment
1	20/63	20/63	20/63	Absent	IVA × 2
2	20/320	20/630	20/320	Absent	IVA × 1
3	20/80	20/63	20/25	Complete	None
4	20/63	20/50	20/20	Complete	IVA × 1
5	20/320	20/200	20/125	Incomplete	IVR × 2
6	20/40	20/25	20/25	Absent	IVA × 1
7	20/50	20/20	20/16	Complete	IVA × 1
8	20/500	20/320	20/500	Absent	IVR × 1
9	20/500	20/2000	20/630	Absent	None
10	20/500	20/250	20/200	Absent	None
11	20/250	20/40	20/25	Complete	IVR × 2
12	20/250	20/200	20/100	Absent	IVR × 1, IVA × 2
13	20/100	20/63	20/63	Complete	None

**Figure 2:**
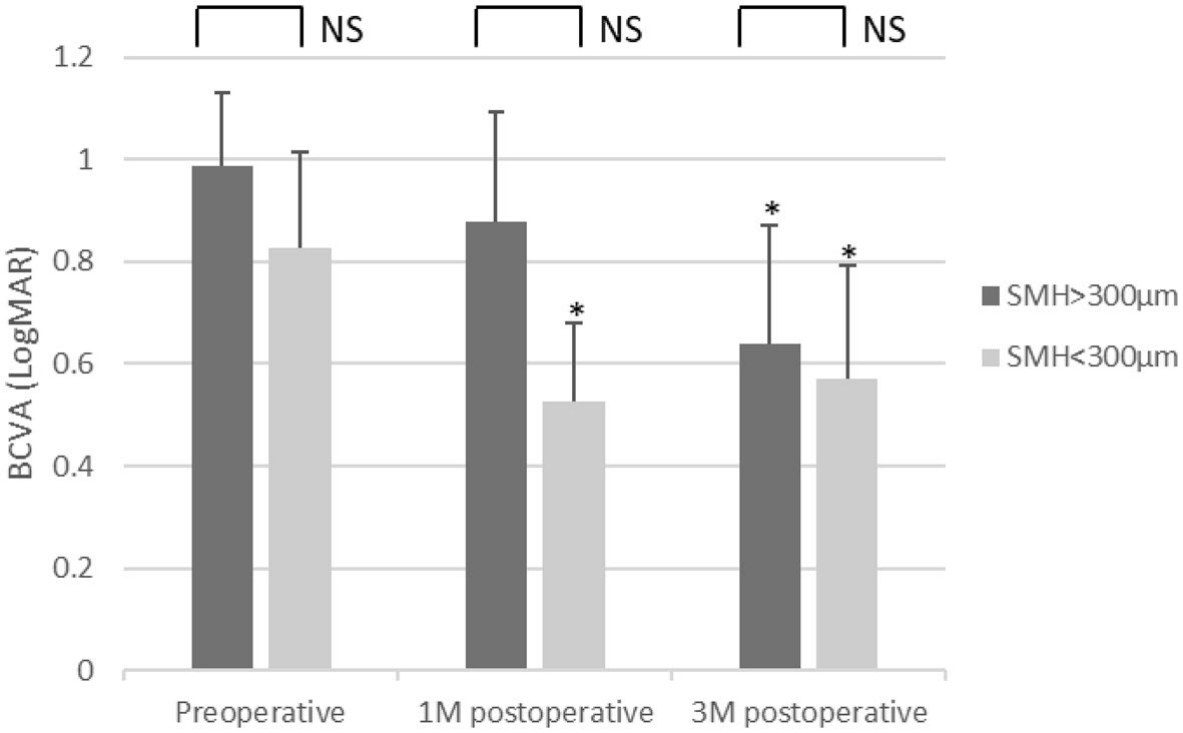
Comparison of postoperative BCVA classified by preoperative SMH height (μm). *P < 0.05.

Division of the 13 eyes into 2 groups by SMH height of 300 µm revealed significant visual improvements as early as 1 month in the SMH < 300 µm group (P < 0.05 at 1 month and 3 months) and at 3 months in the SMH > 300 µm group (P < 0.05). No statistically significant difference in visual acuity was observed between the two groups at all time points (Fig. 2).

**Figure 3:**
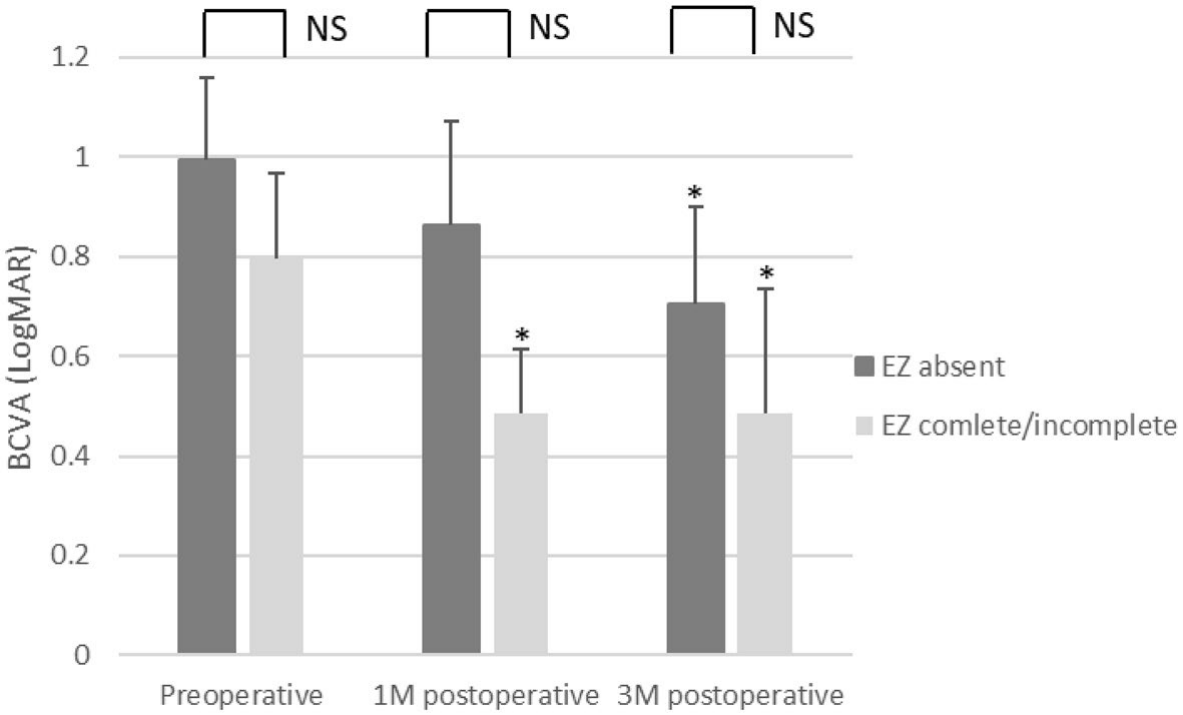
Comparison of postoperative BCVA classified by preoperative EZ status. *P < 0.05.

With regard to EZ, eyes were divided into 2 groups by the presence or absence of preoperatively detectable EZ. Significant visual improvements were observed as early as 1 month in eyes with detectable EZ (P < 0.05 at 1 month and 3 months) and at 3 months in eyes without preoperatively detectable EZ (P < 0.05). No statistically significant difference in visual acuity was observed between the two groups at all time points (Fig. 3). To explore the possibility that the SMH height may affect detection of the EZ status, we evaluate the SMH height of EZ complete/ incomplete group and that of EZ absent group and found the difference was not significant (p = 0.836).

Other possible factors associated with postoperative visual acuity were also analyzed (Table 4). A strong negative correlation was observed between preoperative SMH size and postoperative BCVA at 3 months. A larger SMH size was associated with poorer postoperative BCVA at 3 months (r = 0.689, P < 0.01). With regard to preoperative BCVA, a strong positive correlation was detected with postoperative BCVA both at 1 month (r = 0.837, P < 0.01) and 3 months (r = 0.855, P < 0.01) (Table 4). Preoperative PED height and width were also negatively correlated with postoperative BCVA (PED height: r = 0.577, P < 0.05, PED width: r = 0.672, P < 0.05). Since longer duration of SRH itself has a detrimental effect on visual prognosis^13^, we next sought to analyze preoperative factors that may be associated with postoperative duration required for SMH displacement. However, among factors studied, no significant factor was detected. We also performed a multivariate analysis to search the predicting factor for postoperative BCVA. Analysis included SMH height, disc size of the SMH, PED height, PED width, preoperative number of PED, duration from onset and surgery, postoperative duration until SRH displacement, preoperative BCVA, and the preoperative EZ status. Among these factors, preoperative BCVA was the only significant factor involved in postoperative BCVA at 3 months (Table 5). With regard to the relationship between postoperative EZ status and visual prognosis, we analyzed the difference of postoperative BCVA at 3 months between eyes with and without postoperatively detectable EZ. We observed a significantly better postoperative BCVA in eyes with detectable EZ (P < 0.05).

**Table 4:** Correlation analysis of parameters associated with postoperative duration until SMH displacement and postoperative BCVA at 3 months post-surgery.

	Postoperative duration until SMH displacement (days)	Postoperative BCVA at 3 M (log MAR)
Duration from onset and surgery (days)	r = 0.026	r = 0.317
	p = 0.936	p = 0.316
Postoperative duration until SMH displacement (days)		r = 0.529
		p = 0.870
SMH height (µm)	r = − 0.258	r = − 0.226
	p = 0.418	p = 0.457
SMH size (disc size)	r = 0.278	r = 0.689
	p = 0.382	**p < 0.01**
Preoperative BCVA (log MAR)	r = − 0.162	r = 0.855
	p = 0.616	**p < 0.005**
PED height (µm)	r = 0.015	r = 0.577
	p = 0.963	**p < 0.05**
PED width (µm)	r = 0.399	r = 0.672
	p = 0.199	**p < 0.05**
Preoperative PED number	r = 0.551	r = 0.358
	p = 0.063	p = 0.230
Preoperative EZ status (0: absent, 1: comlete/incomplete)	r = − 0.342	r = − 0.204
	p = 0.277	p = 0.504

Significant values are given in bold.

**Table 5:** Preoperative factors affecting postoperative visual acuity at 3 months.

Variables	Multivariate analysis	
	β value	P value
Duration from onset and surgery (days)	0.096	0.594
Postoperative duration until SMH displacement (days)	− 0.385	0.3
SMH height (µm)	0.954	0.153
SMH size (disc size)	− 0.168	0.457
Preoperative BCVA (log MAR)	1.093	**0.035**
PED height (µm)	− 1.303	0.152
PED width (µm)	1.379	0.106
Preoperative PED number	1.615	0.153
Preoperative EZ status (0: absent, 1: comlete/incomplete)	1.508	0.111

Significant values are given in bold.

### Anatomical outcomes documented on OCT

**Figure 4:**
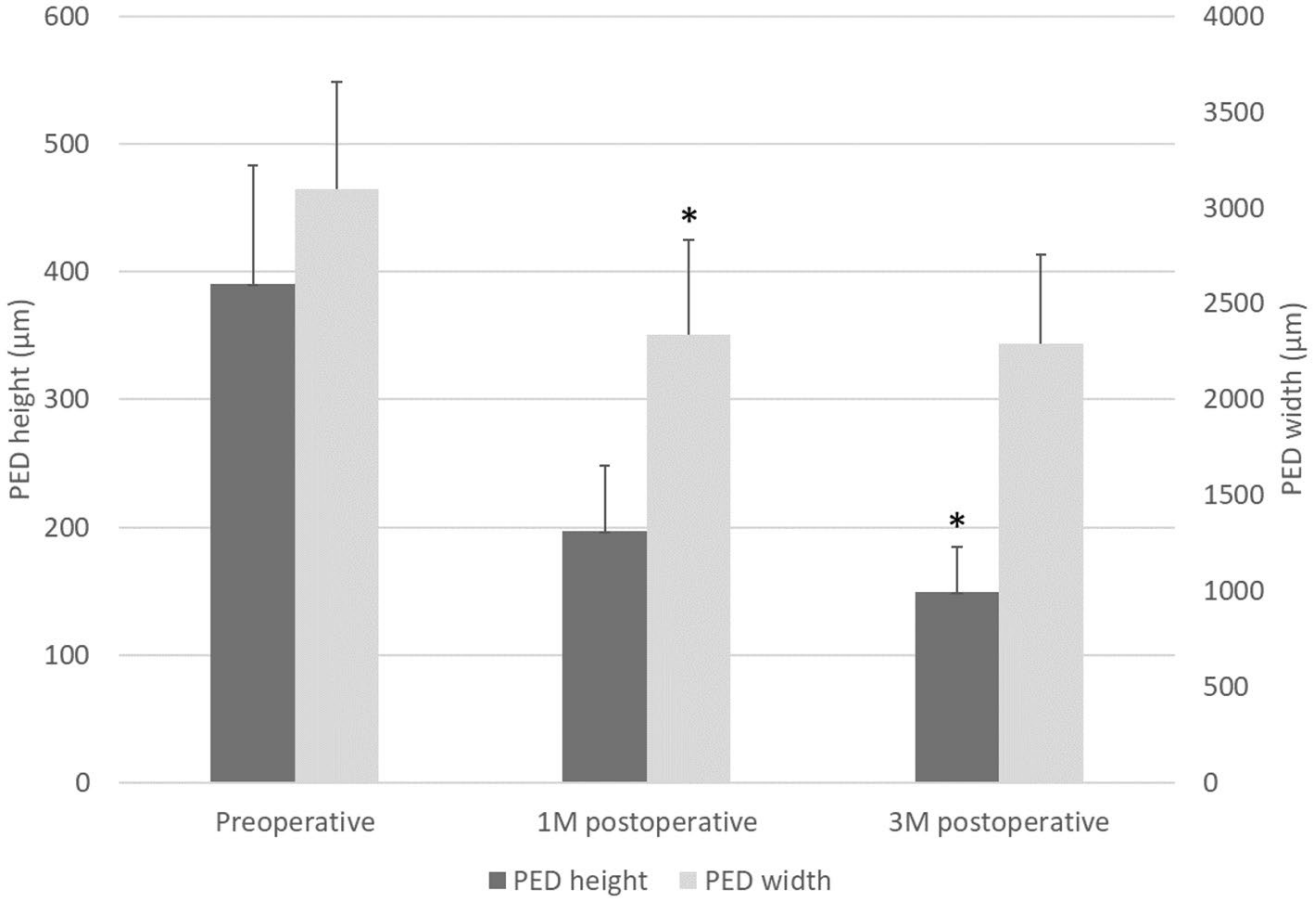
Preoperative and postoperative PED height, width (μm). *P < 0.05.

Complete displacement of subretinal hemorrhage from the foveal center at 1 and 3 months was achieved in 9 (69.2%) and 12 eyes (92.3%), respectively. One eye (patient 2) exhibited incomplete displacement. Postoperative mean height of SMH at 1 and 3 months decreased to 79.8 ± 38.3 µm (P < 0.01 vs. preoperative) and 15.8 ± 16.4 µm (P < 0.01 vs preoperative), respectively. Mean PED height was 196.9 ± 51.3 µm (P = 0.06 vs. preoperative) at 1 month and 149.4 ± 35.0 µm (P < 0.05 vs. preoperative) at 3 months post-surgery. Mean PED width was 2336.6 ± 494.6 µm (P < 0.05 vs. preoperative) at 1 month and 2292.4.1 ± 465.0 µm (P = 0.071 vs. preoperative) at 3 months post-surgery (Fig. 4). PED was observed after surgery in all cases at 3 months. The mean number of PED at 3 months was 1.15 ± 0.10, with no statistically significant change at 3 months after surgery (P = 0.08). Among the factors analyzed, no significant correlation was detected with duration required for SMH displacement (Table 4).

### Surgical complications

No intraoperative complications occurred. Postoperative complications were observed in 3 eyes. One eye presented with recurrent subretinal hemorrhage once (patient 2) and was successfully treated. In the remaining 2 eyes, both eyes first had re-operation for postoperative unclearing vitreous hemorrhage (VH) and then underwent 2nd re-operation for retinal detachment (RD).

## Discussion

SMH secondary to neovascular AMD is associated with poor visual outcomes predominantly due to retinal damage from subretinal clots and progression of underlying AMD. Martel et al.^12^ first reported the surgical technique of PPV with subretinal injection of t-PA, bevacizumab, and air. Air in the subretinal space poses several merits during the healing process. First, it keeps the retina detached for several days, which enables t-PA and hemorrhage to mix and liquefy easily. Second, it creates an air lock to prevent reflux of t-PA, anti-VEGF, and liquefied SMH to the vitreous cavity. In the study by Martel et al., patients were positioned upright after surgery, allowing subretinal air to exert a direct force by pushing the hemorrhage inferiorly. Gas in the vitreous cavity maintains subretinal air in the macula area instead of moving superiorly, which facilitates hemorrhage displacement distant to the fovea^14^. Sharma et al.^14^ employed this technique and reported that complete displacement from the foveal center was achieved in 24/24 eyes (100%). In a study by Kadonosono et al., patients were treated with PPV and subretinal injection of tPA and air without fluid-air exchange. All patients (13/13 eyes) achieved total subfoveal blood displacement^15^. Avci et al.^16^ reported the results of cases treated with PPV and subretinal injection of both t-PA and bevacizumab followed by fluid-air exchange. All patients (30/30 eyes) achieved total or subtotal resolution of SMH. In this study, we evaluated the outcomes of surgical treatment including PPV with subretinal injection of t-PA, air, bevacizumab, and fluid-air exchange. Our results demonstrated that SMH was completely displaced in 12/13 patients (92.3%), which is comparable to previous studies.

In our study, mean preoperative visual acuity was 20/160 (0.90 ± 0.12 in log MAR), which improved significantly to 20/80 (0.60 ± 0.15 in log MAR) at 3 months. Postoperative log MAR at 3 months improved by at least 0.1 log MAR in 9 eyes (69.2%) and worsened in 1 eye (7.7%). The 3 remaining eyes (23.1%) exhibited a change in log MAR of less than 0.1 compared to preoperative BCVA. The only case that exhibited worsened BCVA was due to recurrent SRH. In previous studies using subretinal air injection, BCVA in log MAR changed from 1.95 at baseline to 0.85 at 3 months in the report by Sharma et al.^14^ and from 1.18 to 0.70 in the report by Kadonosono et al.^15^ Although the mean improvement in BCVA (0.30 in log MAR) in our study was not so remarkable as compared to these two studies, it may be at least partly due to a ceiling effect of postoperative BCVA, since preoperative BCVA in our study was the best among the 3 studies. Similarly, in studies performed without subretinal air injection, including our previous study, the BCVA in log MAR improved from 1.40 to 0.86 in the report by Hirashima et al. and from 2.11 to 1.13 in the report by Avci et al.^11,16^. Accordingly, although preoperative BCVA in this study was the best among previously reported values, significant improvements in postoperative BCVA following treatment using subretinal air injection may be expected.

Preoperative SMH duration and BCVA have been identified as predictors of postoperative BCVA^8,13^. Hirashima et al. reported that eyes with preoperative SMH height < 400 µm had significantly better postoperative BCVA compared to eyes with SMH height > 400 µm. In our study, both preoperative log MAR BCVA and SMH size were positively correlated with postoperative log MAR BCVA. With regard to SMH size, Hattenbach et al.^13^ reported that SMH size was not significantly associated with postoperative visual improvements in eyes with a duration of hemorrhage ≤ 14 days. In contrast, Sandhu et al.^17^ reported that eyes with SMH size ≤ 5.5-disc diameters exhibited a trend for better visual outcomes. Regarding preoperative SMH duration, the mean duration in this study was 10.25 ± 2.9 days (12/13 eyes), and no correlation with postoperative BCVA was detected. Further, preoperative SMH height did not significantly affect postoperative BCVA at either 1 or 3 months. However, eyes with SMH < 300 µm exhibited significant BCVA improvements as early as 1 month, suggesting that these eyes tended to have earlier visual improvements. With regard to postoperative remaining SMH, Avci et al. reported a significant difference in postoperative BCVA between groups with total and subtotal SMH displacement. In our study, the mean number of days for SMH displacement after surgery was 11.1 ± 6.3 days, excluding 1 eye which caused a recurrence of SMH within 3 months. Although we found that no significant preoperative factors analyzed were associated with postoperative duration required for SMH displacement, this may be partly due to that the period is an approximate number but not an exact period. Moreover, we evaluated the effects of preoperative EZ on postoperative BCVA. Preoperative EZ has been identified as a key predictor of visual recovery and better postoperative BCVA after surgery for epiretinal membrane^18,19^ and macula hole surgery^20^. Notably, our study is the first to analyze EZ status and visual outcomes after surgery using subretinal air injection for SMH. Although we did not observe a direct correlation between EZ status and postoperative BCVA at any time point, we identified significant improvements that occurred earlier in the group with detectable EZ at baseline. In a study that did not perform subretinal air injection^16^, preoperative SMH area was significantly smaller (P < 0.05) in the patients with preoperative continuous EZ; however, preoperative EZ status had no relationship with postoperative BCVA. In contrast, our previous study without subretinal air and bevacizumab injection demonstrated that postoperative BCVA was significantly better in eyes with preoperative intact EZ. Since the frequency of total SMH displacement in the study by Avci et al. was relatively low (16/30), the discrepancy among these studies may be partly due to differences in anatomical surgical outcomes. Previous studies have shown that postoperative EZ status is associated with visual prognosis in various retinal diseases^21–23^. However, this relationship in SMH secondary to AMD remains to be elucidated. We analyzed the difference of postoperative BCVA at 3 months between eyes with and without postoperatively detectable EZ and observed a significantly better postoperative BCVA in eyes with detectable EZ.

We observed that both PED height and width decreased significantly at follow-up, which is in contrast to our previous report that revealed a significant reduction only in PED height but not in PED width^11^. The additional improvements in PED parameters observed in this study may be due to the surgical techniques employed, such as subretinal injections of both bevacizumab and air. PED is a known risk factor for AMD progression and can lead to eventual vision loss^24,25^. Even with anti-VEGF treatments, PED is associated with worse VA^26^ and resistance to anti-VEGF therapy^27^. However, the relationship between PED and visual prognosis after vitrectomy surgery remains to be evaluated. In our previous study, we did not detect a significant correlation between postoperative BCVA and preoperative features of PED including height, width, and number^11^. In this study, preoperative PED height and width exhibited significant negative correlations with postoperative BCVA. This difference may be at least partly underpinned by the improved anatomical outcomes in terms of reduction in PED parameters observed in this study. Accordingly, when this procedure involves the administration of air and Avastin® under the retina, preoperative PED status may be a predictive factor for visual prognosis. Further studies examining the relationship between PED and postoperative BCVA are warranted.

As subretinal air injection is a relatively novel technique, surgical complications should be considered. In the report by Sharma et al. using PPV with subretinal injection of air and t-PA, SMH (20.8%), vitreous hemorrhage (12.5%), retinal detachment (8.3%), and macular hole (4.2%) were reported as postoperative complications. The surgical procedures in the study by Sharma et al. were similar to ours except that they used filtered air in some cases as a vitreous cavity tamponade. Kadonosono et al. used a specially designed 47-gauge microneedle for subretinal injections without a gas tamponade. In their study, 1 intraoperative macular hole formation (7.7%) and 1 postoperative vitreous hemorrhage (7.7%) occurred, and no case of recurrent SMH was reported. It is possible that a smaller injection needle decreased complications such as vitreous hemorrhage and retinal detachment. In studies without subretinal air injection, similar complications including vitreous hemorrhage, retinal detachment, and SMH were reported^11,16,28^. In our study, the frequency of vitreous hemorrhage (15.4%), retinal detachment (15.4%), and recurrent SMH (7.7%) was comparable to previous reports^14^. We measured SMH size with an optic disc, and the top 3 largest SMH were cases with postoperative complications (patients 2, 8, and 9). This indicates that a large SMH may increase the risk of postoperative complications such as recurrence of SMH, VH, or RD.

In contrast to other studies using multiple techniques or procedures performed by multiple surgeons^28–30^, the operation was performed by a single experienced surgeon and the same technique was applied to all cases to ensure technical uniformity in this study. However, our study has several limitations. The number of cases was relatively small, and the design was retrospective. With regard to evaluation using preoperative OCT findings, the number or size of PEDs may have been underestimated due to a thick overlying SMH. Moreover, the follow-up period for surgical outcomes was relatively short. In this regard, future studies with larger scale and longer follow-up period are warranted to evaluate the effectiveness of this surgical technique.

In summary, our study demonstrates that PPV with subretinal injection of t-PA, air, and bevacizumab can effectively achieve both anatomical and visual improvement. Our results also highlighted several preoperative findings including EZ status and SMH height as factors that have significant influences on the duration required for visual improvements. Preoperative BCVA was identified as the definite factor associated with visual outcomes.

## Methods

### Participants

We reviewed the medical records of 13 patients with SMH secondary to neovascular AMD. In all cases, SMH involved the foveal center. The initial surgical treatments were performed by a single surgeon (H.O.) at Hyogo Prefectural Amagasaki General Medical Center between February 2018 and July 2020. All patients underwent pars plana vitrectomy with subretinal injection of t-PA, bevacizumab, and filtered air, and were followed-up for at least 3 months. The study was approved by Ethics Committee of Hyogo Prefectural Amagasaki General Medical Center, and it was conducted in accordance with the tenets of the Declaration of Helsinki. The study adhered to the study protocol and conducted as per the ethical guidelines. Informed consent was obtained from all patients.

Preoperative examinations included best-corrected visual acuity (5 m Landolt chart), intraocular pressure measurements, OCT, color fundus photography, slit lamp biomicroscopy, and fundus examination. Medical records of the following data were reviewed: age, sex, SMH duration, best-corrected visual acuity, anticoagulant medication, color fundus photography, and OCT. BCVA was converted to log MAR scale for statistical analysis. Total SMH displacement was defined as the absence of hemorrhage in the fovea on color fundus photography and OCT examination at 3 months post-surgery. SMH size was evaluated using color fundus photography and recorded as the ratio to the longest diameter of the optic disc.

### OCT measurement

**Figure 5:**
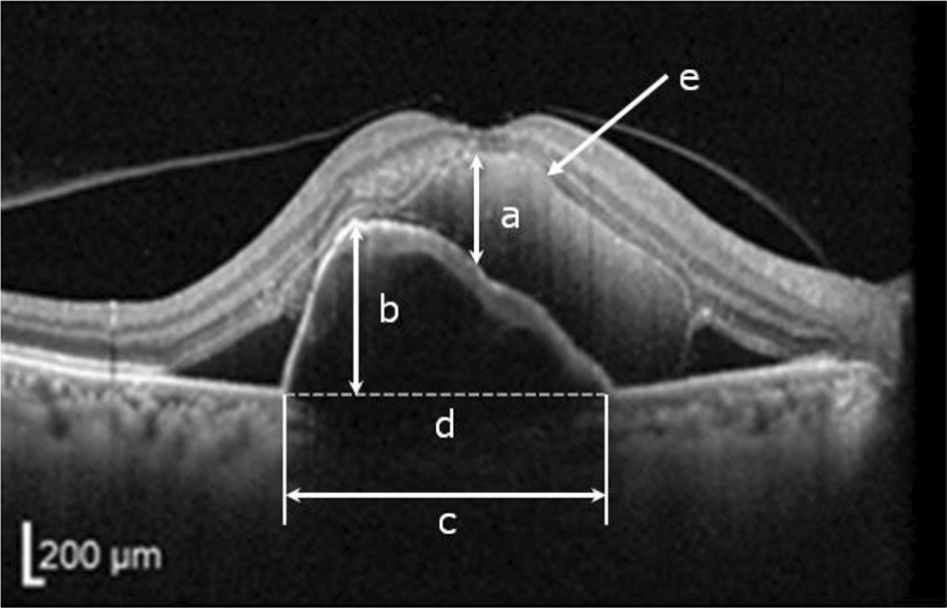
Measurement of preoperative OCT parameters. Preoperative OCT with SMH. (a) SMH height. (b) PED height. (c) PED width. (d) Presumed RPE line. (e) Ellipsoid zone (EZ).

**Figure 6:**
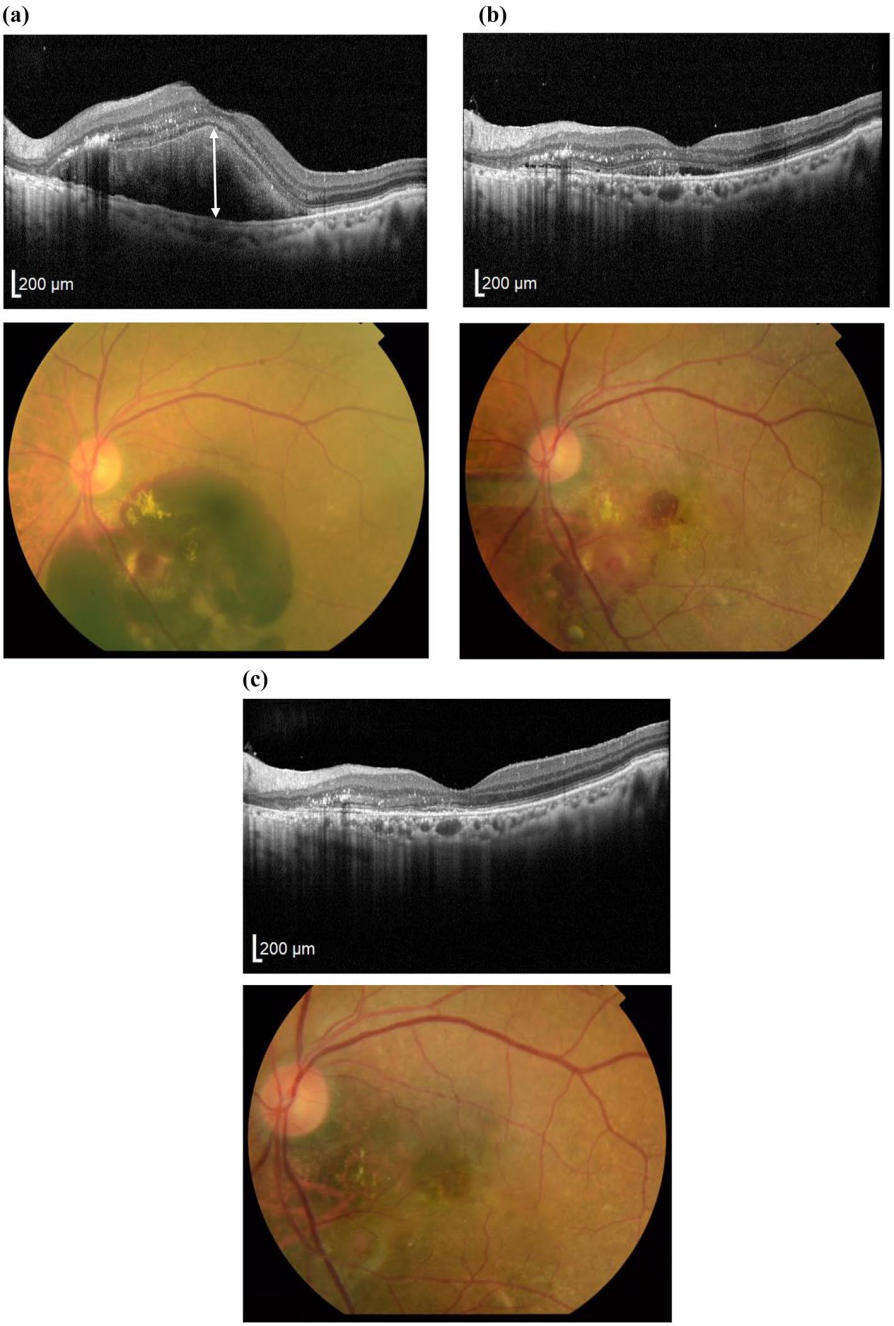
A case of an 83-year-old woman (patient 11). Horizontal OCT images and color fundus photographs at (**a**) baseline, (**b**) 1 month, and (**c**) 3 months post-surgery. Preoperative BCVA was 20/250, and SMH height (double headed arrow) was 604 μm. (**a**) The ellipsoid zone was absent before surgery. (**b**) At 1 month after surgery, SMH decreased to 66 μm. (**c**) At 3 months after surgery, SMH was completely absorbed and BCVA improved to 20/25. The ellipsoid zone was detectable at the fovea.

For OCT, we used the SD-OCT (Spectralis®, Heidelberg Engineering, Inc., MA, USA) protocol for measurements. The protocol consists of 13 horizontal lines followed by vertical lines, each with a 9.2-mm scan length and 127-µm interval (13-line volume scan). Measurements of OCT parameters were performed as described by Hirashima et al^11^ (Figs. 5 and 6). The horizontal OCT scan at the central fovea was used for analyses of OCT parameters including the SMH height, PED height, PED width, and the status of EZ. SMH height was defined as the maximal distance between the inner surfaces of the SMH and retinal pigment epithelium (RPE) at the central fovea. The status of EZ was defined as “absent” in cases showing no detectable EZ, “incomplete” in cases with partly detectable EZ, and “complete” in cases with complete EZ within the central 1 mm. The number of PED was counted using 25 sequential images at a 248-µm interval encompassing an 8.9 mm × 6.0 mm area of the macula. PED height was defined as the maximal distance between the innermost point of the detached RPE and a line connecting both ends of the PED. PED width was defined as the maximal distance between both ends of the detached RPE. When the RPE line was undetectable at the central fovea due to a thick SMH, measurements were performed using a presumed RPE line, defined as a line connecting both ends of the closest detectable RPE lines. SMH displacement was determined by color fundus photography and OCT images. An independent observer (H. O.) reviewed the measurements.

### Interventions

All surgery was performed with a standard 27-gauge vitrectomy system (EVA, DORC, Zuidland, Netherlands or Constellation Vision System, Alcon, Inc, Geneva, Switzerland). After core vitrectomy, posterior vitreous detachment was induced if necessary, followed by peripheral vitrectomy. Using a 38-gauge flexible canula (PolyTip® Cannula 27 g/37 g, MedOne, FL, USA) connected to a 1-mL syringe of the viscous fluid control unit in the vitrectomy machine, 0.2–0.3 mL of t-PA (Cleactor®, Eisai, Tokyo, Japan) 8000 IU/0.1 mL and 0.1 mL bevacizumab (Avastin®, Chugai, Tokyo, Japan) 2.5 µg/0.1 mL were slowly injected into the submacular space. Using the same canula, an additional 0.25–0.3 mL of filtered air was injected into the submacular space from a site near the inferior arcade. Partial fluid-air exchange was performed to replace 60–70% of the vitreous cavity with air. This was performed to utilize fluid buoyance to successfully position the submacular air to the fovea. After surgery, patients were instructed to maintain a supine position for 90 min to facilitate dissolution of SMH by t-PA, followed by a 45° diagonal prone position for several hours to prevent superior tracking of the SMH induced by immediate full prone positioning. Subsequently, patients were requested to maintain a fully prone position.

### Statistical analysis

The results are expressed as the mean ± standard error (SE). Data were analyzed using the Mann–Whitney U test or Wilcoxon signed-rank test to evaluate the differences between two groups. Pearson correlation coefficients was used to estimate the relationship between the potential influencing preoperative factors and BCVA at 3 months. We used multivariate analyses for adjustment of confounding factors. P < 0.05 was considered statistically significant. Statistical analysis was performed with commercially available software (BellCurve for Excel, Social Survey Research Information Co., Tokyo, Japan).

## Data Availability

All relevant data are provided within the manuscript.
